# Supplementary material for: Targeting IL-17A enhances imatinib efficacy in Philadelphia chromosome-positive B-cell acute lymphoblastic leukemia
Source: Nat Commun. 2024 Jan 3;15:203. doi: 10.1038/s41467-023-44270-3 (PMC10764960; doi:10.1038/s41467-023-44270-3)
Supplement: Supplementary file 1 — Supplementary Information [file 41467_2023_44270_MOESM1_ESM.pdf]

Supplementary information

Targeting IL-17A enhances imatinib efficacy in Philadelphia chromosome-positive B-cell acute lymphoblastic leukemia

Feng Wang[1,2#], Yunxuan Li[2#], Zhaona Yang[1,3,4#], Wenbin Cao[5#], Ying Liu[2], Luyao Zhao[2], Tingting Zhang[2], Chenxi Zhao[2], Jinmei Yu[1,4], Jiaojiao Yu[1,4], Jichao Zhou[1,4], Xiaowei Zhang[1,4], Ping-ping Li[1,4], Mingzhe Han[5], Sizhou Feng[5], Billy Wai-Lung Ng[6,7], Zhuo-wei Hu[1,4], Erlie Jiang[5]*, Ke Li[2]* and Bing Cui[1,4]*

Supplementary information includes six Supplementary Figures.

Supplementary Figure 1. P210 *BCR-ABL* induced B-ALL mouse model.

Supplementary Figure 2. IL-17A promotes the homing of Ph^+ B-ALL cells.

Supplementary Figure 3. IL-17A deficiency or neutralization attenuates the homing of Ph^+ B-ALL cells.

Supplementary Figure 4. IL-17A activates BCR-ABL and JAK/STAT3 signaling pathways in Ph^+ B-ALL.

Supplementary Figure 5. CXCL16 promotes the differentiation of Th17 cells.

Supplementary Figure 6. Gating strategies.

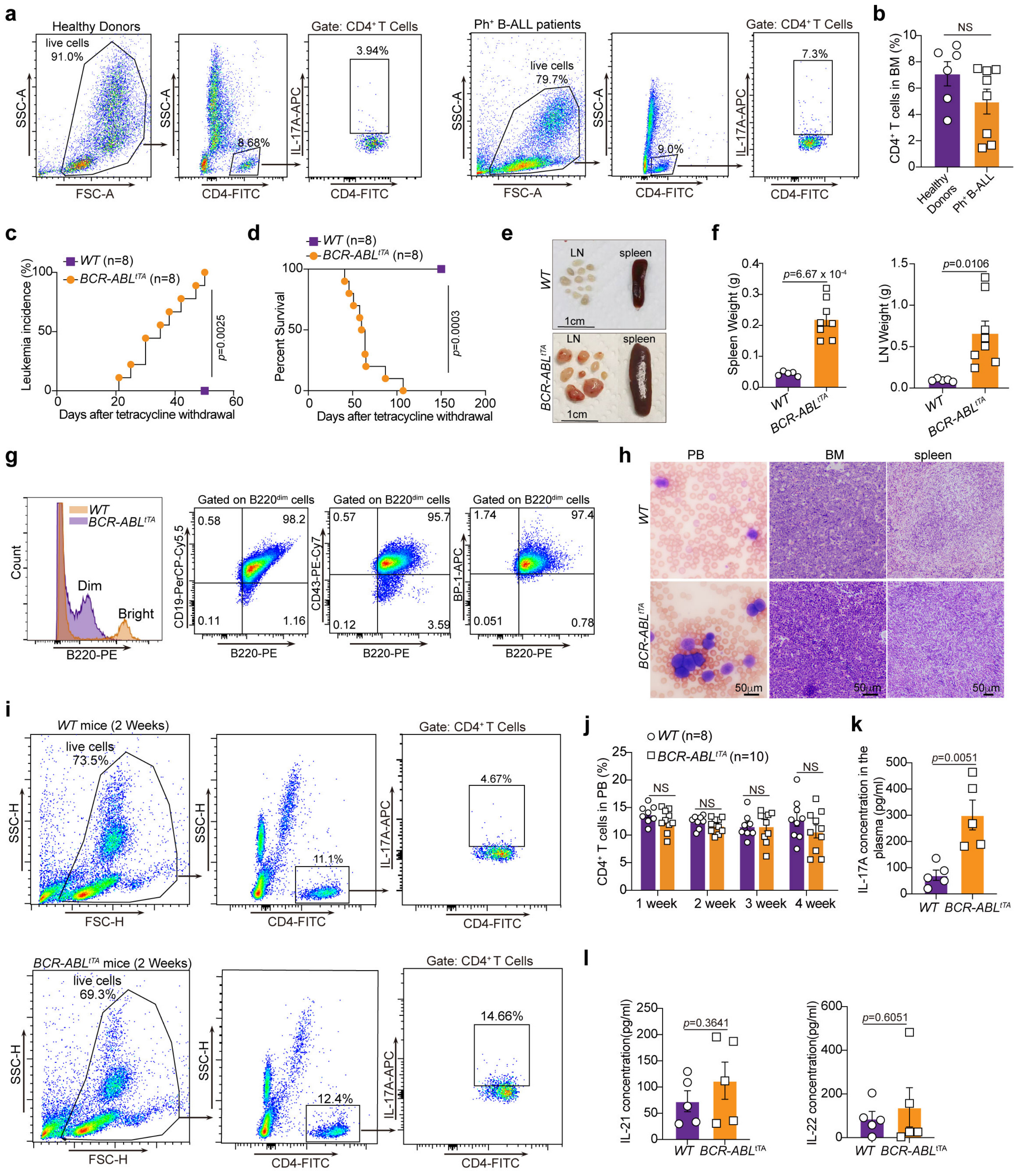

Supplementary Figure 1. P210 *BCR-ABL* induced B-ALL mouse model.

(a) Representative FACS plots to illustrate the gating strategy of Th17 cells in BM samples from Ph$^+$ B-ALL patients and healthy donors were shown. **(b)** Flow cytometric analysis of the percentage of CD4$^+$ T cells in the BM of Ph$^+$ B-ALL patients (n = 8 samples) and healthy donors (n = 6 samples). The gating strategy for CD4$^+$

T cells is shown in Supplementary Figure 1a. **(c-d)** Incidence of B-ALL **(c)** and Kaplan-Meier survival curves**(d)** of *WT* mice and *BCR-ABL*^{tTA} mice after induction of *BCR–ABL1* expression by tetracycline withdrawal (n = 8 mice per group). **(e-f)** Representative images of spleens and LNs **(e)** and spleen and LN weights in the *WT* mice and *BCR-ABL*^{tTA} mice after induction of BCR–ABL1 expression by tetracycline withdrawal for 1-3 months (n = 8 mice per group). **(g)** Left: Representative FACS histogram of spleen cells isolated from *WT* and diseased *BCR-ABL*^{tTA} mice demonstrating expression of B220. Lymphoblasts from diseased *BCR-ABL*^{tTA} mice are B220^{dim}. Right: Representative FACS plots for detecting the coexpression of B220, CD19, CD43 and BP-1 in B220^{dim} spleen cells of *BCR-ABL*^{tTA} mice (n = 3 mice per group). **(h)** Representative images of Wright-Giemsa-stained peripheral blood smear and H&E staining of spleen and BM from *BCR-ABL*^{tTA} mice after withdrawal of tetracycline administration for 50 days (n = 3 mice per group). **(i)** The gating strategy for Th17 cells in PB samples of *WT* mice or *BCR-ABL*^{tTA} -transplanted mice at two weeks is shown. **(j)** Flow cytometric analysis of the percentage of CD4$^+$ T cells in PB of *WT* mice (n = 8 mice) or *BCR-ABL*^{tTA} -transplanted mice (n = 10 mice) at the indicated time. The gating strategy for CD4$^+$ T cells is shown in Supplementary Figure 1i. **(k-l)** The IL-17A **(k)**, IL-21 and IL-22 **(l)** levels in the plasma of *BCR-ABL*^{tTA} mice and *WT* mice (n=5 mice per group) were quantified by a Mouse TH17 Cytokines Array. Statistical significance was calculated by **(b, f, j, k, l)** two-tailed Student's t-test; **(c, d)** two-sided log-rank test. Data are presented as means ± S.E.M. Source data are provided as a Source Data file.

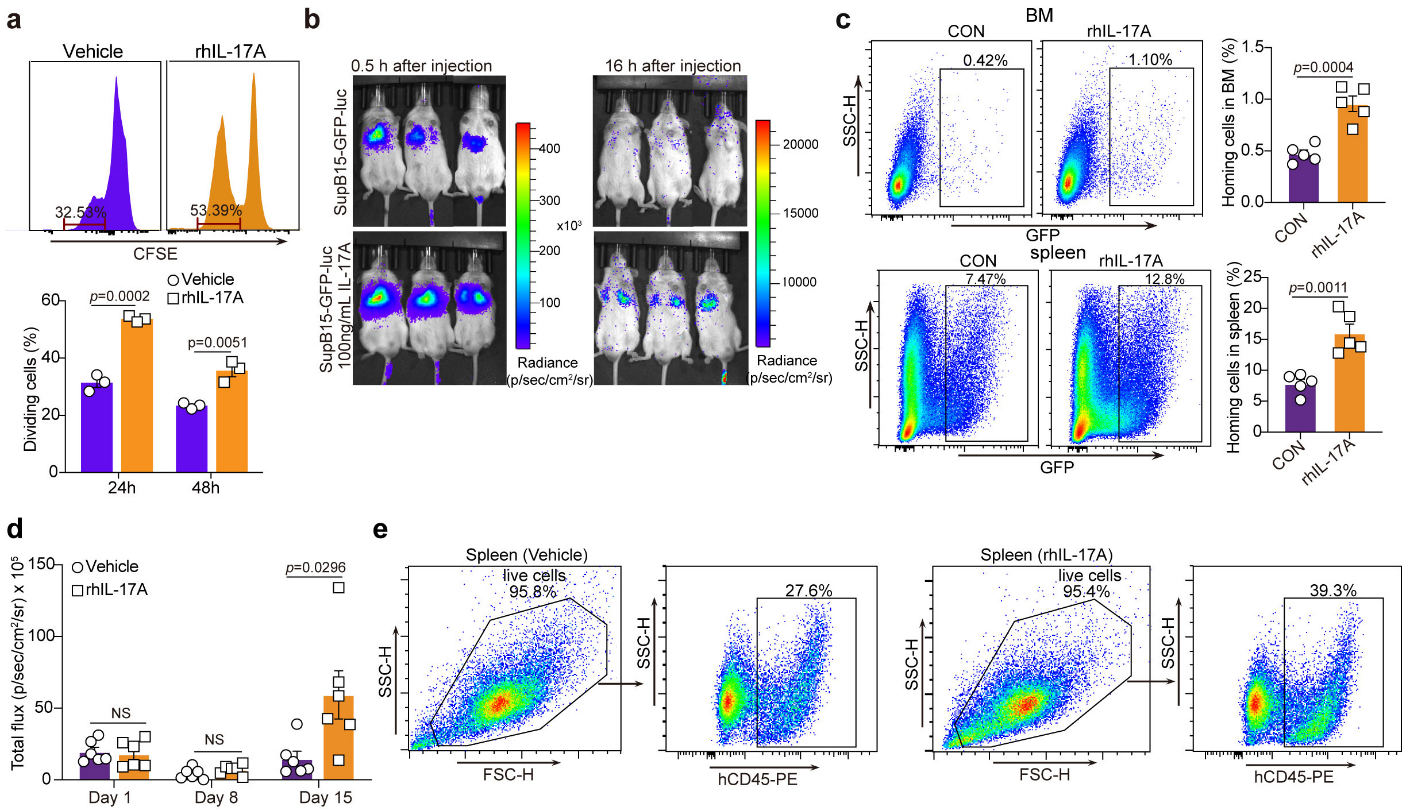

Supplementary Figure 2. IL-17A promotes the homing of Ph$^+$ B-ALL cells.

(a) The cell division of primary Ph$^+$ B-ALL cells incubated with rhIL-17A (100 ng/ml) or PBS control for indicated times was analyzed by CFSE staining. The statistical analysis for the percentage of dividing cells are shown at the bottom. **(b)** The infiltration of B-ALL cells *in vivo* was quantitatively monitored by the biophotonic imaging system in NSG mice xenografted with GFP-luc tagged SupB15 cells with or without 100ng/mL rhIL-17A treatment. The infiltration of B-ALL cells was monitored at 0.5 h and 16 h after transplantation. **(c)** Representative FACS plots and quantification of the percentage of homed B-ALL cells with or without IL17A treatment in the spleen and BM of recipient mice at 16 h after transplantation (n=5 mice per group). **(d)** The effect of rhIL-17A treatment on the progression of B-ALL was monitored by the biophotonic imaging system. The total flux of indicated mice was determined by Living Image software (Perkin Elmer) (n=6 mice per group). **(e)** Representative FACS plots to illustrate the gating strategy for hCD45$^+$ cells in the spleens of SupB15-GFP-LUC cell-engrafted NSG mice treated with rhIL-17 or vehicle (n = 5 mice per group). **(a)** n = 3 independent

experiments. Statistical significance was calculated by **(a, c, d)** two-tailed Student's t-test; Data are presented as means ± S.E.M. Source data are provided as a Source Data file.

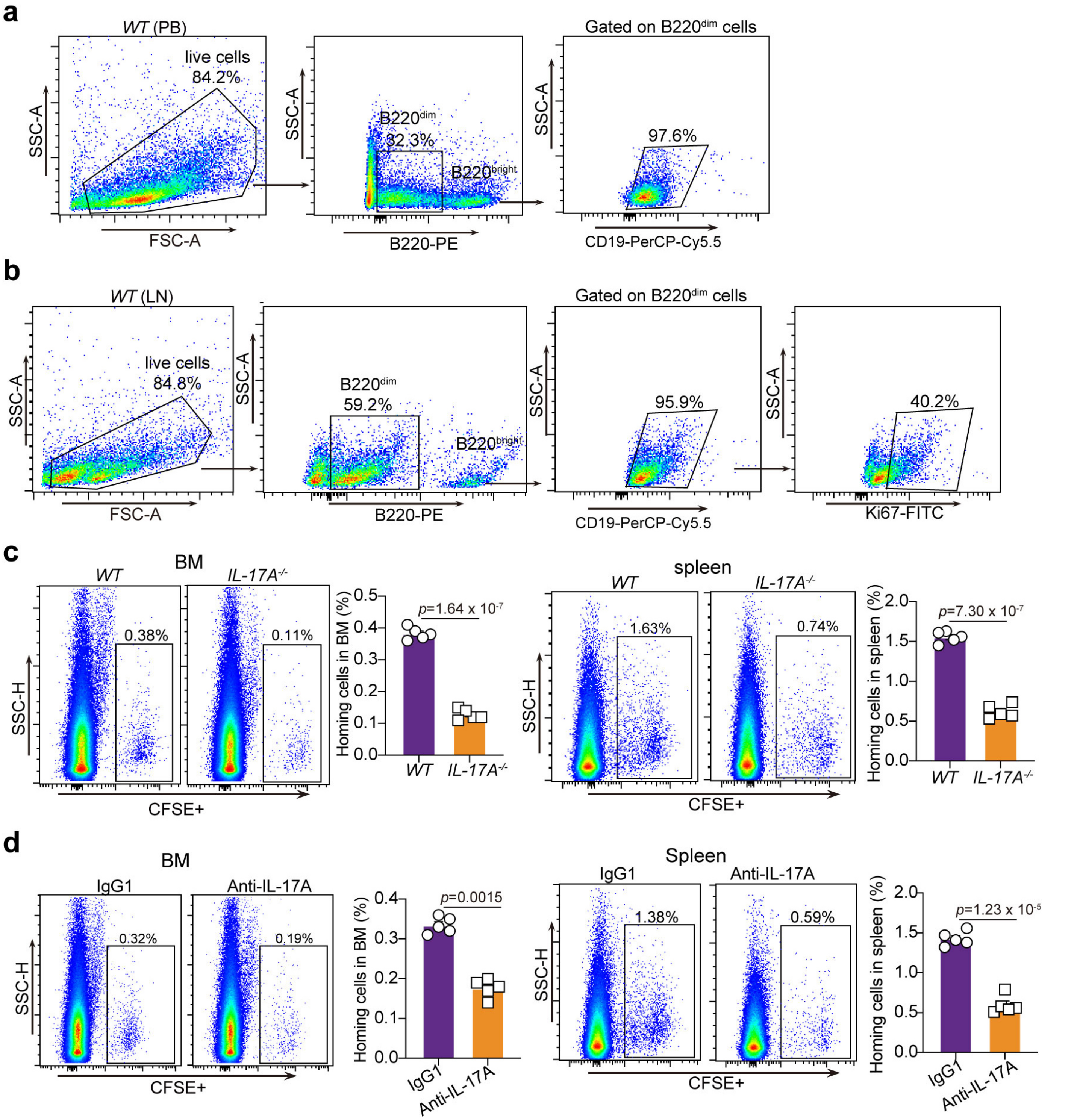

Supplementary Figure 3. IL-17A deficiency or neutralization attenuates the homing of Ph⁺ B-ALL cells. **(a)** Representative FACS plots to illustrate the gating strategy for B220^{dim}CD19^{+} cells in the PB of *WT* mice with primary B-ALL cells secondary transplantation at two weeks (n=5 mice per group). **(b)** Representative FACS plots to illustrate the gating strategy for B220^{dim} CD19^{+} Ki-67^{+}cells in the LN of *WT* mice with primary B-ALL cells secondary transplantation (n=5 mice per group). **(c)** Representative FACS plots and quantification

of the percentage of homed B-ALL cells in the spleen and BM of WT mice and *IL-17A*$^{-/-}$ mice at 16 h after transplantation (n=5 mice per group). Data are represented as means ± S.E.M. Statistical significance was determined by a two-tailed Student's t-test. **(d)** Representative FACS plots and quantification of the percentage of homed B-ALL cells with or without anti-IL17A treatment in the spleen and BM of recipient mice at 16 h after transplantation (n=5 mice per group). Data are represented as means ± S.E.M. Statistical significance was determined by a two-tailed Student's t-test. Source data are provided as a Source Data file.

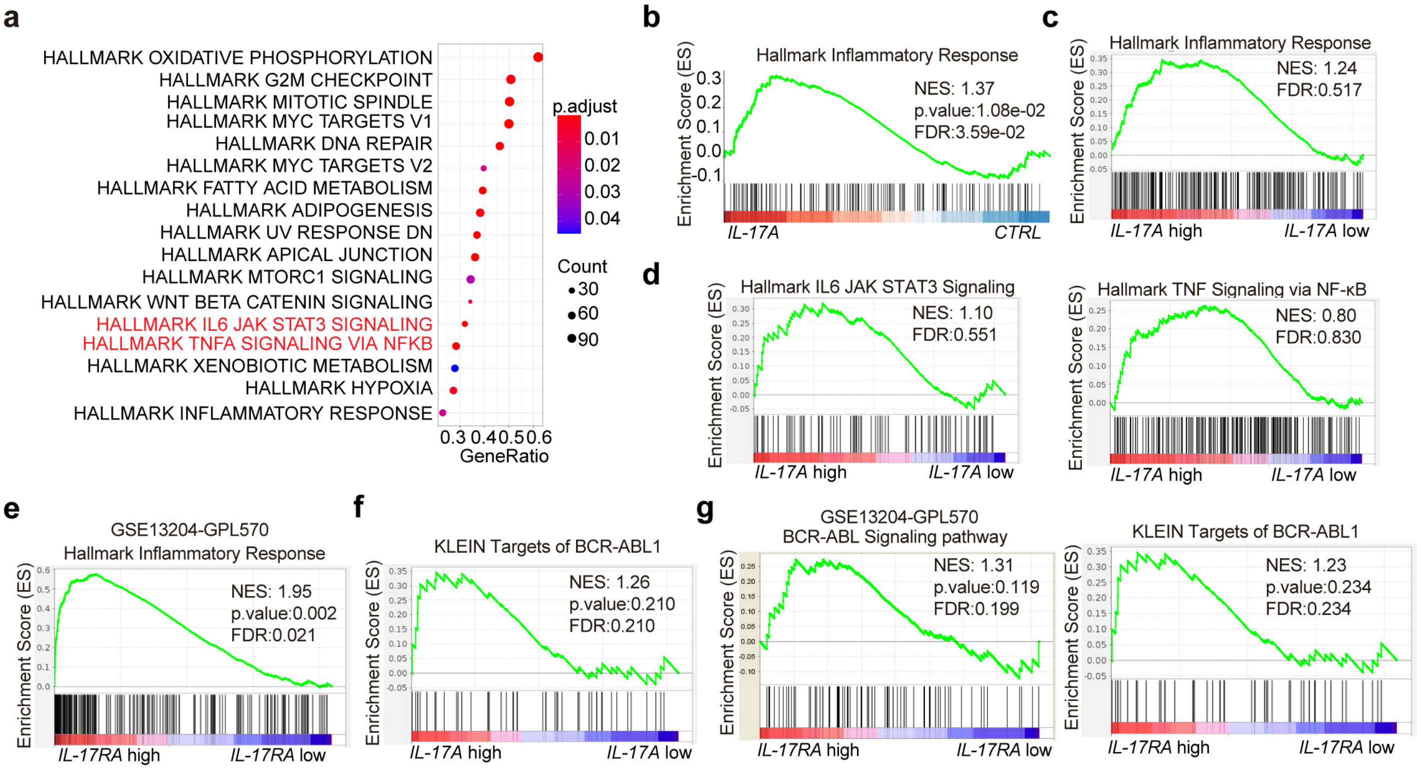

Supplementary Figure 4. IL-17A activates BCR-ABL and JAK/STAT3 signaling pathways in Ph$^+$ B-ALL. **(a)** Molecular signature classification screening by GSEA analysis with top enriched Hallmark gene sets in primary mouse leukemia cells treated with rmIL-17A treatment or PBS control (CON). NES, normalized enrichment score. FDR, false discovery rate. **(b)** Molecular signature classification screening by GSEA analysis with top enriched inflammatory response in primary mouse leukemia cells treated with rmIL-17A treatment or PBS control (*CTRL*). **(c-d)** GSEA demonstrating the enrichment of gene sets related to an inflammatory response **(c),** human and human IL6 JAK STAT3 Signaling pathways **(d, left)** and TNF signaling via NF-kB **(d, right)** in Ph$^+$ B-ALL patients expressing *IL-17A* mRNA (205707_at) above the median level (*IL-17A* high) (n=61), with patients with leukemia expressing *IL-17A* mRNA below the median level (*IL-17A* low) (n=61) from the GSE13204 dataset. **(e)** GSEA demonstrating the enrichment of gene sets related to an inflammatory response in Ph$^+$ B-ALL patients expressing high *IL-17RA* mRNA (*IL-1RA* high: above the median level) vs low *IL-17RA* mRNA *(IL-17RA* low: below the median level) from the GSE13204 dataset. **(f-g)** GSEA demonstrating the enrichment of gene sets related to KLEIN targets of BCR-ABL and BCR-ABL signaling pathway in Ph$^+$ B-ALL patients expressing high *IL-17A* mRNA (above the median level)

vs low *IL-17A* mRNA (below the median level) **(f)** or high *IL-17RA* mRNA (above the median level) vs low *IL-17RA* mRNA (below the median level) **(g)** from the GSE13204 dataset. **(a-g)** Statistical significance was determined by a one-sided permutation test, and statistical adjustments were made for multiple comparisons. Source data are provided as a Source Data file.

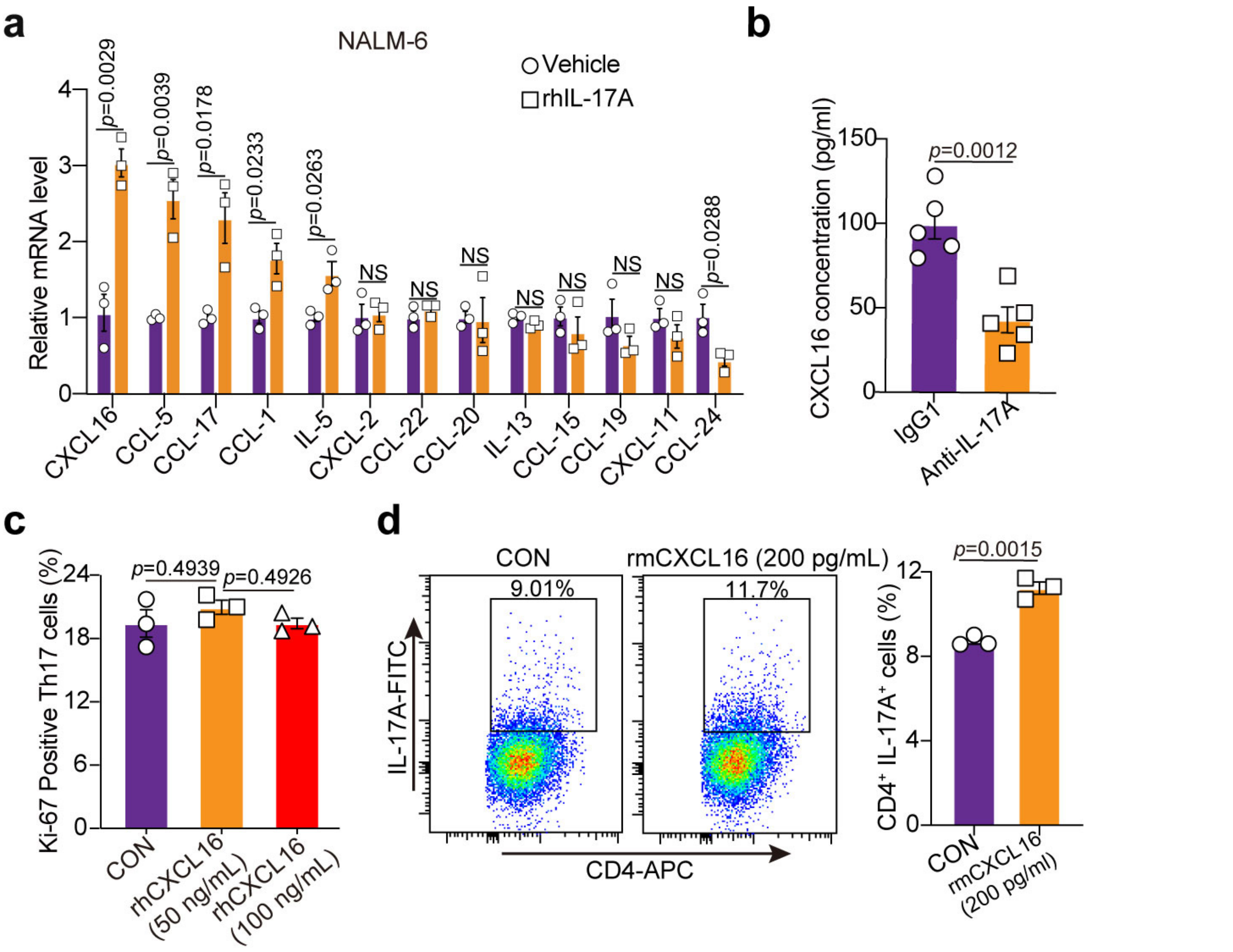

Supplementary Figure 5. CXCL16 promotes the differentiation of Th17 cells.

(a) Real-time PCR analysis of the relative mRNA level of chemokines genes in NALM-6 cells treated with rhIL-17A (100 ng/ml) or PBS control for 24 h. **(b)** The CXCL16 levels in the serum of C57BL/6 mice transplanted with 1 x 10^5 primary mouse B-ALL cells isolated from spleens of *BCR-ABL*^{tTA} mice treated with or without anti-IL-17A neutralizing antibody for 3 weeks were measured by ELISA (n=5 mice per group). **(c)** The effect of CXCL16 on the proliferation activity of Th17 cells was determined by Ki-67 staining. Human Th17 cells were sorted from PBMC of healthy donors and cultured with or without 50ng/mL or 100ng/mL rhCXCL16 treatment for 24 h, and the proliferation activity of Th17 cells was analyzed by flow cytometry. **(d)** Flow cytometric analysis of the percentage of Th17 cells in naïve $CD4^+$ T cells with or without 200pg/mL rmCXCL16 treatment. Representative FACS plots and statistical analysis for the percentage of Th17 cells are shown. **(a, c, d)** n = 3

independent experiments. Statistical significance was calculated by **(a, b, d)** two-tailed Student's t-test; **(c)** one-way ANOVA with Tukey's multiple comparison tests; Data are presented as means ± S.E.M. Source data are provided as a Source Data file.

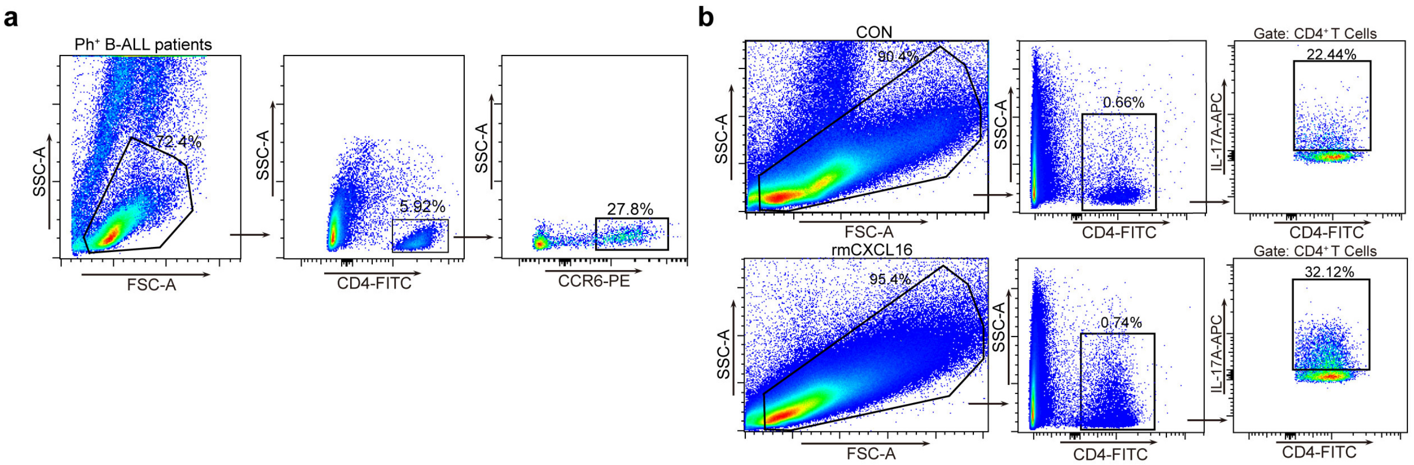

Supplementary Figure 6. Gating strategies. (a) The strategy for isolating Th17 cells from PB or BM of Ph$^+$ B-ALL patients. Single-cell suspensions were obtained from the BM of human Ph$^+$ B-ALL patients and labeled with human CD4 antibody and human CCR6 antibody. Then, CD4$^+$ CCR6$^+$ cells were sorted by FACS Aria III cell sorter. **(b)** Representative FACS plots to illustrate the gating strategy for Th17 cells in the CD4$^+$ T cells in the BM of mice with secondary transplantation treated with or without rmCXCL16 (n = 6 mice per group). Source data are provided as a Source Data file.
