# Supplementary material for: Targeting IL-17A enhances imatinib efficacy in Philadelphia chromosome-positive B-cell acute lymphoblastic leukemia
Source: Nat Commun. 2024 Jan 3;15:203. doi: 10.1038/s41467-023-44270-3 (PMC10764960; doi:10.1038/s41467-023-44270-3)
Supplement: Supplementary file 3 — Description of Additional Supplementary Files [file 41467_2023_44270_MOESM3_ESM.pdf]

Description of Additional Supplementary Files

Supplementary Data 1| Clinical characteristics of patients (15 males and 10 Females) between the ages of 12 and 51 in this study.
