# Supplementary material for: Targeting IL-17A enhances imatinib efficacy in Philadelphia chromosome-positive B-cell acute lymphoblastic leukemia
Source: Nat Commun. 2024 Jan 3;15:203. doi: 10.1038/s41467-023-44270-3 (PMC10764960; doi:10.1038/s41467-023-44270-3)
Supplement: Supplementary file 5 — Reporting Summary [file 41467_2023_44270_MOESM5_ESM.pdf]

nature portfolio

Corresponding author(s): Bing Cui, Ke Li & Erlie Jiang

Last updated by author(s): Nov 18, 2023

Reporting Summary

Nature Portfolio wishes to improve the reproducibility of the work that we publish. This form provides structure for consistency and transparency in reporting. For further information on Nature Portfolio policies, see our Editorial Policies and the Editorial Policy Checklist.

Statistics

For all statistical analyses, confirm that the following items are present in the figure legend, table legend, main text, or Methods section.

n/a	Confirmed	
☐	☒	The exact sample size (*n*) for each experimental group/condition, given as a discrete number and unit of measurement
☐	☒	A statement on whether measurements were taken from distinct samples or whether the same sample was measured repeatedly
☐	☒	The statistical test(s) used AND whether they are one- or two-sided<br>*Only common tests should be described solely by name; describe more complex techniques in the Methods section.*
☒	☐	A description of all covariates tested
☐	☒	A description of any assumptions or corrections, such as tests of normality and adjustment for multiple comparisons
☐	☒	A full description of the statistical parameters including central tendency (e.g. means) or other basic estimates (e.g. regression coefficient) AND variation (e.g. standard deviation) or associated estimates of uncertainty (e.g. confidence intervals)
☐	☒	For null hypothesis testing, the test statistic (e.g. *F*, *t*, *r*) with confidence intervals, effect sizes, degrees of freedom and *P* value noted<br>*Give P values as exact values whenever suitable.*
☒	☐	For Bayesian analysis, information on the choice of priors and Markov chain Monte Carlo settings
☒	☐	For hierarchical and complex designs, identification of the appropriate level for tests and full reporting of outcomes
☐	☒	Estimates of effect sizes (e.g. Cohen's *d*, Pearson's *r*), indicating how they were calculated

Our web collection on statistics for biologists contains articles on many of the points above.

Software and code

Policy information about availability of computer code

Data collection	Standard software and the respective analysis tools provided by manufacturers were listed in the methods (Yena qTOWER3G PCR, Tanon 5200 chemiluminescent imaging system, Olympus DP72 microscope, Olympus Microsystems (Fv3000), BD FACS Verse, CytoFLEX flow cytometer. Standard software and the respective analysis tools provided by manufacturers were listed in the methods (Yena qTOWER3G PCR, Tanon 5200 chemiluminescent imaging system, Olympus DP72 microscope, Olympus Microsystems (Fv3000), BD FACS Verse, CytoFLEX flow cytometer, IVIS SpectrumCT In Vivo Imaging System.
Data analysis	Student’s t-test, one-way ANOVA analysis and Kaplan-Meier analysis were done by using Prism GraphPad 9.0. All flow cytometry data were analyzed using FCS Express 6 or FlowJo 10.8.1 software. Western blots images were analyzed by Image J. Quantitative image analysis was performed with Imaris 9.3.1 software. GSEA was performed using the clusterProfiler package by R 4.0.3.The bioluminescence imaging data was analyzed with the Living Image Software (version 3.2).

For manuscripts utilizing custom algorithms or software that are central to the research but not yet described in published literature, software must be made available to editors and reviewers. We strongly encourage code deposition in a community repository (e.g. GitHub). See the Nature Portfolio guidelines for submitting code & software for further information.

Data

Policy information about availability of data

All manuscripts must include a data availability statement. This statement should provide the following information, where applicable:

- Accession codes, unique identifiers, or web links for publicly available datasets
- A description of any restrictions on data availability
- For clinical datasets or third party data, please ensure that the statement adheres to our policy

The RNA-seq data generated in this study have been deposited in the NCBI Gene Expression Omnibus (GEO) database under accession code GSE210091. The single cell datasets from HDs and Ph+ B-ALL patients was analyzed on the following accession code GSE134759. The overall survival (OS) and disease-free survival (DFS) of patients with high IL-17A and in low IL-17A expression were analyzed in data set GSE11877. IL-17 RA and IL-17 RC expression in Ph+ B-ALL patients, Ph- B-ALL patients and Healthy Donors and enrichment of gene sets in Ph+ B-ALL patients with IL-17 RA high vs IL-17 RA low or IL-17 A high vs IL-17 A low were analyzed on the following accession code GSE13204. The uncropped blot figures, and original data underlying Figs. 1–8 and Supplementary Figs. 1–6 are provided as a Source Data file. The remaining data are available within the Article, Supplementary Information or Source Data file. Source data are provided with this paper.

Research involving human participants, their data, or biological material

Policy information about studies with human participants or human data. See also policy information about sex, gender (identity/presentation), and sexual orientation and race, ethnicity and racism.

Reporting on sex and gender	Our analysis did not focus on identifying sex/gender-specific differences. We used human B-ALL patient specimens to isolate primary leukemia cell regardless of sex/gender.
Reporting on race, ethnicity, or other socially relevant groupings	The researchers are not reporting on race, ethnicity, or other socially relevant groupings. However, for ethnicity, both Han ethic and non-Han ethnic participants were recruited.; for race, all participants were chinese.
Population characteristics	Human B-ALL patient specimens and healthy donors were collected from patients with pathologically confirmed B-ALL at the Institute of Hematology and Blood Diseases Hospital of PUMC. The patients (age between 12 and 51, 15 males and 10 Females) were Chinese with average age 28 years old.
Recruitment	Patients (age between 12 and 51, 15 males and 10 Females) were enrolled in by their oncologist at Institute of Hematology and Blood Diseases Hospital of PUMC. BM and PBMC specimens were collected from patients with informed consent (IIT2021011-EC-1).
Ethics oversight	The procedure was approved by the institutional review board at the Ethics Committee of the Institute of Hematology and Blood Diseases Hospital of PUMC.

Note that full information on the approval of the study protocol must also be provided in the manuscript.

Field-specific reporting

Please select the one below that is the best fit for your research. If you are not sure, read the appropriate sections before making your selection.

[x] Life sciences [ ] Behavioural & social sciences [ ] Ecological, evolutionary & environmental sciences

For a reference copy of the document with all sections, see nature.com/documents/nr-reporting-summary-flat.pdf

Life sciences study design

All studies must disclose on these points even when the disclosure is negative.

Sample size	For in vitro experiments, at least three biological replicates were achieved for most of the experiments. Such sample sizes are typical for the in vitro experiments and sufficient for a statistical analysis. Generally, we used n ≥ 5 mice per genotype and condition. For in vivo experiments, a sample size of n =3-8 mice was used per experimental group. Sample size was determined based on our previous experience (Cancer Cell. 2017;31(5):697#710; Nat Commun. 2020;11 (1):6361; Sci Transl Med. 2021; 13 (586): eabb2914;), which is sufficient to generate statistically significant results. No statistical method was used to predetermine sample size.
Data exclusions	No data were excluded from the analysis.
Replication	Most of the in vitro experiments were repeated independently at least three times except for those specifically indicated in the figure legends. Multiple mice (n = 3-8/group) were used for every in vivo experiment. All the attempts at replication were successful.
Randomization	For animal studies, the mice were earmarked before grouping and then were randomly separated into groups by an independent person; however, no particular method of randomization was used. For other experiments, cells/samples were randomly assigned to groups to avoid bias.
Blinding	All experiments were performed in a non-blinded manner, because the experimental design was complicated, the researchers were limited, and blinding feasibility was poor.

Reporting for specific materials, systems and methods

We require information from authors about some types of materials, experimental systems and methods used in many studies. Here, indicate whether each material, system or method listed is relevant to your study. If you are not sure if a list item applies to your research, read the appropriate section before selecting a response.

Materials & experimental systems

n/a	Involved in the study
	☒ Antibodies
	☒ Eukaryotic cell lines
☒	☐ Palaeontology and archaeology
	☒ Animals and other organisms
☒	☐ Clinical data
☒	☐ Dual use research of concern
☒	☐ Plants

Methods

n/a	Involved in the study
☒	☐ ChIP-seq
	☒ Flow cytometry
☒	☐ MRI-based neuroimaging

Antibodies

Antibodies used

Immunoblotting : anti-NF-kappaB p65 (D14E12) antibody (Cell Signaling Technology(CST), #8242 1:1000), anti-phospho-NF-kappaB p65 (Ser536) (93H1) antibody (Cell Signaling Technology(CST), #3033, 1:1000), anti-BCR antibody (Cell Signaling Technology(CST), 3902S, 1:1000), anti-phospho-Bcr (Tyr177) antibody (Cell Signaling Technology(CST), #3901, 1:1000), anti-Stat5 (D2O6Y) antibody (Cell Signaling Technology(CST), #94205, 1:1000), anti-phospho-Stat5 (Tyr694) (D47E7) antibody (Cell Signaling Technology(CST), #4322, 1:1000), anti-Stat3 (D1B2J) antibody (Cell Signaling Technology(CST), #30835, 1:1000), anti-phospho-Stat3 (Tyr705) (D3A7) antibody (Cell Signaling Technology(CST), #9145, 1:1000), anti-AKT (pan) (11E7) antibody (Cell Signaling Technology(CST), #4685, 1:1000), anti-phospho-Akt (Ser473) (D9E) antibody (Cell Signaling Technology(CST), #4060, 1:1000), anti-MEK1/2 (D1A5) antibody (CST, #8727, 1:1000), anti-phospho- MEK1/2 (Ser217/221) (41G9) (CST, #9154, 1:1000), anti-Histone H3 (D1H2) XP (CST, # 60932, 1:1000), anti-β-Actin (8H10D10) antibody (Cell Signaling Technology(CST), #3700, 1:1000), and anti-GAPDH (ZSGB-BIO,TA-08, OT12D9, 1:2000). For immunofluorescence staining: anti-mouse B220/CD45R antibody [RA3-6B2] (R&D, MAB1217, 1:100), anti-CD4 [EPR19514] antibody (Abcam, ab183685, 1:100), anti-IL-17A [4K5F6]antibody (Abcam, ab189377, 1:100), anti-CXCL16 antibody (R&D, AF503, 1:100). Alexa Fluor 488 (Thermo Fisher, R37114, 1:200), Alexa Fluor 555 (Thermo Fisher, A31572, 1:200), Alexa Fluor 647 (Thermo Fisher, A21247, 1:200), Alexa Fluor 647 (Thermo Fisher, A21447, 1:200), Alexa Fluor 555 (Thermo Fisher, A21434, 1:200). For flow cytometry: PE anti-mouse/human CD45R/B220 antibody (BioLegend, RA3-6B2, 103208, 1:100), APC/Cyanine7 anti-mouse CD19 antibody (BioLegend, 1D3/CD19, 152412, 1:100), APC anti-mouse CD4 antibidy (BioLegend, RM4-4, 116014, 1:100), FITC anti-mouse IL-17A antibody (BioLegend, TC11-18H10.1, 506907, 1:100), PE/Cyanine7 anti-mouse Ki-67 antibody (BioLegend, 16A8, 652426, 1:100), PerCP anti-mouse CD19 Antibody (BioLegend, 6D5, 115531, 1:100), PE anti-human CD19 antibody (BioLegend, HIB19, 302254, 1:100), FITC anti-human CD4 antibody (BioLegend, A161A1, 357405, 1:100), APC anti-human CD4 antibody (BioLegend, OKT4, 317416, 1:100), FITC anti-mouse/human Ki-67 antibody (BioLegend, 11F6, 151212, 1:100), PE/Cyanine7 anti-human IL-17A antibody (BioLegend, BL168, 512315, 1:100), Alexa Fluor® 647 anti-human IL-17A antibody (BioLegend, BL168, 512310, 1:100),human IL-17RA/IL-17R APC-conjugated antibody (R & D, # 133617, FAB177A, 1:100), human IL-17RC Alexa Fluor® 488 antibody (R & D, # 309822, FAB22691,1:100).For Th17 cells enrichment: FITC anti-human CD4 antibody (BioLegend, A161A1, 357405, 1:100) and PE/Cyanine7 anti-human CD196 (CCR6) antibody (BioLegend, G034E3, 353417, 1:100). For in vivo treatment: anti-IL-17A neutralizing antibody (Bio X Cell, 17F3, BP0173 ,5 mg/kg), anti-mouse CXCL16 neutralizing antibody (R&D, # 142417, MAB503, 0.5 mg/kg).

Validation

All antibodies are obtained from commercial sources, and validation was available from the websites of respective vendors; anti-NF-kappaB p65 (D14E12) antibody,human and mouse, WB and IF, (https://www.cellsignal.cn/products/primary-antibodies/nf-kb-p65-d14e12-xp-rabbit-mab/8242);anti-phospho-NF-kappaB p65 (93H1) antibody, human and mouse, WB and IF, (https://www.cellsignal.cn/products/primary-antibodies/phospho-nf-kb-p65-ser536-93h1-rabbit-mab/3033?site-search-type=Products&N=4294956287&Ntt=anti-phospho-nf-kappab+p65+%2893h1%29+antibody&fromPage=plp); anti-BCR antibody, human and mouse, WB, (https://www.cellsignal.cn/products/primary-antibodies/bcr-antibody/3902?site-search-type=Products&N=4294956287&Ntt=3902s%2C&fromPage=plp&_requestid=828213); anti-Phospho-Bcr (Tyr177) antibody, human and mouse, WB, (https://www.cellsignal.cn/products/primary-antibodies/phospho-bcr-tyr177-antibody/3901?site-search-type=Products&N=4294956287&Ntt=%233901&fromPage=plp&_requestid=825684); anti-Stat5 (D2O6Y) antibody , human and mouse, WB, (https://www.cellsignal.cn/products/primary-antibodies/stat5-d3n2b-rabbit-mab/25656?site-search-type=Products&N=4294956287&Ntt=anti-stat5+%28d3n2b%29+antibody&fromPage=plp&_requestid=826122); anti-phospho-Stat5 (Tyr694) (D47E7) antibody, human and mouse, WB, (https://www.cellsignal.cn/products/primary-antibodies/phospho-stat5-tyr694-d47e7-xp-rabbit-mab/4322?site-search-type=Products&N=4294956287&Ntt=anti-phospho-stat5+%28tyr694%29+%28d47e7%29+antibody&fromPage=plp); anti-Stat3 (D1B2J) antibody, human and mouse, WB, (https://www.cellsignal.cn/products/primary-antibodies/stat3-d1b2j-rabbit-mab/30835?site-search-type=Products&N=4294956287&Ntt=anti-stat3+%28d1b2j%29+antibody&fromPage=plp); anti-phospho-Stat3 (Tyr705) (D3A7) antibody,human and mouse, WB, (https://www.cellsignal.cn/products/primary-antibodies/phospho-stat3-tyr705-d3a7-xp-rabbit-mab/9145?site-search-type=Products&N=4294956287&Ntt=anti-phospho-stat3+%28tyr705%29+%28d3a7%29+antibody&fromPage=plp); anti-AKT (pan) (11E7) antibody, human and mouse, WB, (https://www.cellsignal.cn/products/primary-antibodies/akt-pan-11e7-rabbit-mab/4685?site-search-type=Products&N=4294956287&Ntt=anti-akt+antibody&fromPage=plp); anti-phospho-Akt (Ser473) (D9E) antibody, human and mouse, WB, (https://www.cellsignal.cn/products/primary-antibodies/phospho-akt-ser473-d9e-xp-rabbit-mab/4060?site-search-type=Products&N=4294956287&Ntt=anti-phospho-akt+%28ser473%29+%28d9e%29+antibody&fromPage=plp); anti-MEK1/2 antibody, human and mouse, WB, IF, F, (https://www.cellsignal.cn/products/primary-antibodies/mek1-2-d1a5-rabbit-mab/8727?site-search-type=Products&N=4294956287&Ntt=anti-mek1%2F2+%28d1a5%29+antibody&fromPage=plp); anti-Phospho-MEK1/2

(Ser217/221)(41G9) antibody, human and mouse, WB and IP, (https://www.cellsignal.cn/products/primary-antibodies/phospho-mek1-2-ser217-221-41g9-rabbit-mab/9154?site-search-type=Products&N=4294956287&Ntt=anti-phospho-+mek1%2F2+%28ser217%2F221%29&fromPage=plp); anti-Histone H3 (D1H2) XP antibody, human and mouse, WB, IHC, IF, and F, (https://www.cellsignal.cn/products/primary-antibodies/histone-h3-d1h2-xp-rabbit-mab/4499?site-search-type=Products&N=4294956287&Ntt=anti-histone+h3+%28d1h2%29+xp&fromPage=plp); anti-β-Actin (8H10D10) antibody, human and mouse, WB, (https://www.cellsignal.cn/products/primary-antibodies/b-actin-8h10d10-mouse-mab/3700?site-search-type=Products&N=4294956287&Ntt=anti-β-actin+%288h10d10%29+antibody&fromPage=plp); anti-GAPDH, human and mouse, WB, (http://www.zsbio.com/product/TA-08); For immunofluorescence staining: anti-mouse B220/CD45R antibody, mouse, IF, (https://www.rndsystems.com/cn/products/mouse-b220-cd45r-antibody-ra3-6b2_mab1217); anti-CD4 antibody, mouse, IHC-Fr, (https://www.abcam.cn/cd4-antibody-epr19514-ab183685.html); anti-IL-17A antibody, human and mouse, WB and IHC-P, (https://www.abcam.cn/il-17a-antibody-4k5f6-ab189377.html); anti-CXCL16 antibody, mouse, WB and CyTOF, (https://www.rndsystems.com/cn/products/mouse-cxcl16-antibody_af503); Alexa Fluor 488, all, IF, (https://www.thermofisher.cn/cn/zh/antibody/product/Donkey-anti-Mouse-IgG-H-L-Secondary-Antibody-Polyclonal/R37114); Alexa Fluor 555, all, IF, (https://www.thermofisher.com/antibody/product/Donkey-anti-Rabbit-IgG-H-L-Highly-Cross-Adsorbed-Secondary-Antibody-Polyclonal/A-31572);Alexa Fluor 647, all, IF, (https://www.thermofisher.cn/cn/zh/antibody/product/Goat-anti-Rat-IgG-H-L-Cross-Adsorbed-Secondary-Antibody-Polyclonal/A-21247); Alexa Fluor 647, all, IF, (https://www.thermofisher.cn/cn/zh/antibody/product/Donkey-anti-Goat-IgG-H-L-Cross-Adsorbed-Secondary-Antibody-Polyclonal/A-21447); Alexa Fluor 555, all, IF,(https://www.thermofisher.cn/cn/zh/antibody/product/Goat-anti-Rat-IgG-H-L-Cross-Adsorbed-Secondary-Antibody-Polyclonal/A-21434); For flow cytometry: PE anti-mouse/human CD45R/B220 antibody, human and mouse, IF, (https://www.biolegend.com/en-us/products/pe-anti-mouse-human-cd45r-b220-antibody-447); APC/Cyanine7 anti-mouse CD19 antibody, mouse, IF, (https://www.biolegend.com/en-us/products/apc-cyanine7-anti-mouse-cd19-antibody-22018); APC anti-mouse CD4 antibidy, mouse, IF, (https://www.biolegend.com/en-us/products/apc-anti-mouse-cd4-antibody-9224); FITC anti-mouse IL-17A antibody, mouse, IF, (https://www.biolegend.com/en-us/products/fitc-anti-mouse-il-17a-antibody-3534); PE/Cyanine7 anti-mouse Ki-67 antibody, mouse, IF, (https://www.biolegend.com/en-us/products/pe-cyanine7-anti-mouse-ki-67-antibody-13821); PerCP anti-mouse CD19 antibody, mouse, IF, (https://www.biolegend.com/en-us/products/percp-anti-mouse-cd19-antibody-4260); PE anti-human CD19 antibody, human, IF, (https://www.biolegend.com/en-us/products/pe-anti-human-cd19-antibody-719); FITC anti-human CD4 antibody, human, IF, (https://www.biolegend.com/en-us/products/fitc-anti-human-cd4-antibody-8738); APC anti-human CD4 antibody, human, IF, (https://www.biolegend.com/en-us/products/apc-anti-human-cd4-antibody-3657); FITC anti-mouse/human Ki-67 antibody, human and mouse, IF, (https://www.biolegend.com/en-us/products/fitc-anti-mouse-human-ki-67-antibody-18405); PE/Cyanine7 anti-human IL-17A antibody, human, IF, (https://www.biolegend.com/en-us/products/pe-cyanine7-anti-human-il-17a-antibody-5954); Alexa Fluor® 647 anti-human IL-17A antibody, human, IF, (https://www.biolegend.com/en-us/products/alexa-fluor-647-anti-human-il-17a-antibody-4454?GroupID=BLG5454); human IL-17RA/IL-17R APC-conjugated antibody, human, IF, (https://www.rndsystems.com/cn/products/human-il-17ra-il-17r-apc-conjugated-antibody-133617_fab177a); human IL-17RC Alexa Fluor® 488 antibody, human, IF, (https://www.rndsystems.com/cn/products/human-il-17rc-alexa-fluor-488-antibody-309822_fab22691g); For Th17 cells enrichment:FITC anti-human CD4 antibody, human, IF, (https://www.biolegend.com/en-us/products/fitc-anti-human-cd4-antibody-8738); PE/Cyanine7 anti-human CD196 (CCR6) antibody, human, IF, (https://www.biolegend.com/en-us/products/pe-cyanine7-anti-human-cd196-ccr6-antibody-7564); For in vivo treatment: anti-IL-17A neutralizing antibody, mouse, Neutralisation, (https://shop.bio-connect.nl/antibodies/invivoplus-anti-mouse-il-17a/bp0173_5mg/sfid/8780942), anti-mouse CXCL16 neutralizing antibody, mouse, Neutralisation, (https://www.rndsystems.com/cn/products/mouse-cxcl16-antibody_af503).

Eukaryotic cell lines

Policy information about cell lines and Sex and Gender in Research

Cell line source(s)	The acute B-cell leukemia cell lines SupB15, BV173, and NALM-6 were purchased from Cell Resource Center, Peking Union Medical College.
Authentication	All the cell lines were recently authenticated by STR profiling.
Mycoplasma contamination	All cell lines were verified negative for mycoplasma contamination by MycoAlertTM Mycoplasma Detection Kit (Lonza, LT07-318).
Commonly misidentified lines (See ICLAC register)	No commonly misidentified cell lines were used.

Animals and other research organisms

Policy information about studies involving animals; ARRIVE guidelines recommended for reporting animal research, and Sex and Gender in Research

Laboratory animals	NOD-SCID IL2Rg-null (NSG) mice (6-8 weeks old, male) were purchased from the Nanjing Biomedical Research Institute of Nanjing University (Nanjing, China). The tetO-BCR/ABL1 (B6.FVB/N-Tg(tetO-BCR/ABL1)2Dgt/Nju) mice (5–6 weeks old, 1 male and 2 females, strain #N00005) were purchased from the Nanjing Biomedical Research Institute of Nanjing University (Nanjing, China). These mice had been backcrossed over 10 generations onto the C57BL/6 background. MMTV-tTA (B6.Cg-Tg(MMTVtTA)1Mam/J) mice (6–8 weeks old, 2 males and 2 females, Strain #002618) were purchased from The Jackson Laboratory (CA, USA). C57BL/6J mice (female, 6-8 weeks old) were purchased from Hua Fu Kang Technology Co., Ltd. (Beijing, China). BCR-ABLtTA mice were generated by crossing female tetO-BCR/ABL1 (B6.FVB/N-Tg(tetO-BCR/ABL1)2Dgt/Nju) mice with male MMTV-tTA mice (B6.Cg-Tg(MMTVtTA)1Mam/J) under continuous administration of tetracycline (0.5 g/L) in the drinking water. Withdrawal of tetracycline in BCR-ABLtTA mice resulted in the development of Ph+ B-ALL within 1.5-2 months35. IL-17A-/- (C57BL/6Smoc-IL17aem1Smoc) mice (5–6 weeks old, 1 male and 2 females, Cat. NO. NM-KO-00131) were obtained from Shanghai Model Organisms Center, Inc.. To determine the role of IL-17A in leukemogenesis, founder lines of BCR-ABLtTA mice that had features of human B-ALL and scored positive for Ph+ B-ALL were used. A total of 1 x 105 mouse B-ALL-like spleen cells from BCR-ABLtTA mice were intravenously injected into nonirradiated IL-17A-/- mice or WT mice at 8-10 weeks of age. All mice were maintained in the animal facility at the Institute of Materia Medica under specific-

	pathogen-free (SPF) conditions. All mice were maintained in the animal facility at the Institute of Materia Medica under specific-pathogen-free (SPF) conditions. Mice were housed in groups of 4–6 in individually ventilated cages on a 12 hr light/dark cycle (07:30–19:30 light, 19:30–07:30 dark) in a room with controlled temperature (23 ± 2°C) and relative humidity (40–50%). Animals were monitored daily. If mice manifested symptoms such as failure to thrive, weight loss>10% of total body weight, open skin lesions, bleeding, infection, and/or fatigue, they were immediately killed immediately.
Wild animals	The study did not involve wild animals.
Reporting on sex	Sex was not considered in this study.
Field-collected samples	No field-collected samples were used in this study.
Ethics oversight	All animal procedures were conducted according to the guidelines of the Institutional Committee for the Ethics of Animal Care and Treatment of Chinese Academy of Medical Sciences (CAMS) and Peking Union Medical College (PUMC). All animal procedures were consistent with the ARRIVE guidelines.

Note that full information on the approval of the study protocol must also be provided in the manuscript.

Plants

Seed stocks	N/A
Novel plant genotypes	N/A
Authentication	N/A

Flow Cytometry

Plots

Confirm that:

- [x] The axis labels state the marker and fluorochrome used (e.g. CD4-FITC).
- [x] The axis scales are clearly visible. Include numbers along axes only for bottom left plot of group (a 'group' is an analysis of identical markers).
- [x] All plots are contour plots with outliers or pseudocolor plots.
- [x] A numerical value for number of cells or percentage (with statistics) is provided.

Methodology

Sample preparation	About 1*10^6 single cells from PB, spleen, BM, and LN of relevant mice or primary Ph+ B-ALL patients were suspended in PBS and incubated with relevant antibodies for 30 min at room temperature .
Instrument	Data was acquired using BD FACS Verse or CytoFLEX flow cytometer.
Software	Data were analyzed with FCS EXPRESS or FlowJo 10.8.1 software.
Cell population abundance	Cell suspension was isolated from BM or PB of Ph+ B-ALL patients. Then the cell suspension was lysed by a red blood cell lysis buffer to remove red cells and passed through a 40um cell strainer.Th17 cells were sorted with a FITC anti-human CD4 antibody (BioLegend, 357405, 1:100) and a PE/Cyanine7 anti-human CD196 (CCR6) antibody (BioLegend, 353417, 1:100). B-ALL cells were sorted by using MAC CD19 MicroBeads (Miltenyi Biotec, 130-050-301).
Gating strategy	The gating strategies were shown as follow: FSC-A/SSC-A was used to gate live cells, then the CD4+ and IL17A+ cells were gated or FSC-A/SSC-A was used to gate live cells, then the B220dim CD19+ or B220dim CD19+ Ki-67+ cells were gated.The gating strategies for Th17 cells were shown in the Supplementary Fig 1a, Supplementary Fig 1i and Supplementary Fig 6b. The gating strategies for B220dim CD19+ and B220dim CD19+ Ki-67+ cells were shown in the Supplementary Fig 3a and Supplementary Fig 3b. The gating strategies for sorting Th17 cells were shown in Supplementary Fig 6a.

- [x] Tick this box to confirm that a figure exemplifying the gating strategy is provided in the Supplementary Information.
